# COVID-19 preventives consumed in South Africa versus other Sub-Saharan African countries

**DOI:** 10.4102/hsag.v27i0.2061

**Published:** 2022-11-30

**Authors:** Eridiong O. Onyenweaku, Alex K. Tchuenchieu, Hema Kesa

**Affiliations:** 1 Food Evolution Research Laboratory, School of Tourism and Hospitality, College of Business and Economics, University of Johannesburg, Johannesburg, South Africa; 2 Centre for Food and Nutrition Research, Institute of Medical Research and Medicinal Plants Studies, Yaounde, Cameroon

**Keywords:** preventive, foods and drinks, COVID-19, South Africa, sub-Saharan Africa, immune-boosters

## Abstract

**Background:**

The current coronavirus disease 2019 (COVID-19) pandemic has been of global concern as it has affected the health of many and the economies of nations. In order to strengthen the immune system against COVID-19, certain plant-source foods were consumed.

**Aim:**

This study was designed to identify and compare various special foods and drinks consumed to prevent COVID-19 during the lockdown in various sub-Saharan countries in comparison to South Africa (SA), as well as highlighting some current dietary recommendations.

**Setting:**

Online cross-sectional survey in six African countries, namely South Africa, Cameroon, Nigeria, Ghana, Ethiopia and Kenya.

**Methods:**

After sample size determination, an online questionnaire was designed and content validated. The survey link was pretested on 25 people and then circulated for 6 weeks during total lockdown. The proportion of responses for each question were reported using descriptive statistics.

**Results:**

Half of the 817 participants surveyed were not consuming anything special for COVID-19 prevention. South Africans mostly reported the consumption of supplements or conventional medicines (mainly vitamin C and zinc) while for other countries, a variety of natural foods and drinks were mentioned – some having already proved helpful in boosting immune systems. They included infusions of spices with or without honey, fruits and vegetables, medicinal drinks and local beverages.

**Conclusion:**

Programmes and campaigns designed to increase awareness of dietary measures for COVID-19 prevention have proved beneficial and should be promoted. Analytical evaluation of the nutritional and health benefits and antiviral potentials of the identified special foods would help in determining which foods to prioritise and promote in the fight against COVID-19.

**Contribution:**

This study shows the possibility of finding dietary solutions for managing the pandemic and ‘preventive’ potentials of certain plant substances.

## Introduction

The coronavirus disease 2019 (COVID-19) pandemic is currently posing a threat to the health and well-being of humanity. It has emerged as a serious health crisis; hence, it is imperative to develop measures to reduce the spread of the virus. Coronavirus disease 2019 was first reported by the World Health Organization (WHO) on 31 December 2019 (Zhou et al. 2020). The WHO declared the COVID-19 outbreak to be a ‘public health emergency of international concern (PHEIC)’ on 30 January 2020 and announced that countries should take immediate and definite steps to stop it from spreading (Li et al. 2020). There are several clinical symptoms that can occur with COVID-19 infection, such as fatigue, dry cough, fever, dyspnoea and myalgia; in some severe cases, there could be severe pneumonia, kidney failure and acute respiratory syndrome or even death (Abdelhafiz et al. 2020). As of 15 November 2020, the total number of COVID-19 cases confirmed worldwide was 53 976 457, with 1 311 942 deaths and 34 772 744 recoveries (WHO 2020). Following the strict lockdown, it became necessary for government to ease the restrictions but not without protocols in place as measures to decrease the spread of the virus.

Vaccines have been developed, but they are not 100% efficient, and they are not always available or weakly accepted in some parts of the world, such as in Africa (Nachega et al. 2021; Olliaro, Torreele & Vaillant 2021). As a result, it is highly recommended to practise preventive measures such as hand washing, face masks and sanitisers. In contrast to predictions, Africa appears as a continent where the mortality rate due to COVID-19 is relatively low (Lawal 2021; Tcheutchoua et al. 2020). The exception in the sub-Saharan Africa (SSA) region came from South Africa, which has been greatly affected. This country, which is still classified as an emerging country, faced its third COVID-19 wave with still a high mortality rate (2 836 773 cases for 84 152 deaths as of 09 September 2021) (Worldometer 2021). One could therefore question what the difference in its population practices could be compared to the other countries from the region, and such differences include a highly mixed population, dietary choices and tourism. A study by Elnadi et al. (2021), which was carried out in Africa, reported a significant disparity in knowledge, attitude and practice regarding the virus when compared to other continents. Similar studies conducted in SSA found that residents were not very compliant with WHO and national health department recommendations for control measures. They cited ignorance, misinformation and the lack of adequate health facilities or equipment as some of the reasons for this (Akalu, Ayelign & Molla 2020).

Despite these disadvantages, the COVID-19 morbidity and mortality rates reported by African countries seem relatively lower than those reported by outside countries (Tcheutchoua et al. 2020). The main determinant of health is optimal nutrition, which can improve well-being, reduce the risk of several chronic illnesses (such as hypertension, diabetes, cancer and obesity), boost mental and psychological health and prevent fatigue (Flaskerud 2015; US Department of Health and Human Services & US Department of Agriculture 2015). Maintaining good health throughout the lifespan requires ‘nutritional modulation of the immune system’. Infants and young children can benefit from breastfeeding during early childhood because of the presence of antibodies, enzymes and hormones present in breastmilk which protect them against infections (Hoddinott, Tappin & Wright 2008). Similarly, a change in dietary habits and food intake can cause significant effects on the immune system and inflammation response, particularly in the elderly (the more vulnerable group at risk for COVID-19 infection), and this is termed ‘immunosenescence’ and ‘inflammaging’ (Weyh, Kruger & Strasser 2020). Anti-inflammatory effects and increased resistance to upper respiratory tract infections have been linked to certain micronutrients, such as vitamin C, vitamin D, zinc, omega-3 polyunsaturated fatty acids and probiotics (Weyh et al. 2020).

Considering that African populations have a traditional medicine culture used to boost their immunity (Abdullahi 2011), this study aims at determining the unique foods and beverages consumed by distinct African populations in order to reduce the rate of COVID-19 infections during the lockdown. It also seeks to evaluate the preventive properties that these special foods and drinks may possess, while highlighting current dietary suggestions for reducing the rate of COVID-19 infections.

## Research methodology

### Study design: Online cross-sectional survey

#### Setting

The country of South Africa is a multiracial one (Asian-, black-, mixed race-, Indian-, and white people) with people from various cultures all over the world such as Indian, Pakistani, Dutch and West African cultures. The other five sub-Saharan countries of Cameroon, Nigeria, Ghana, Ethiopia and Kenya are also not only quite popular but well-populated. The countries were chosen for the study based on their unique population and the availability of field workers for data collection. South Africa recorded high COVID-19 infections and death rates, and this singled it out in this research as a basis for comparison.

### Sample population and technique

The study was conducted via an online survey over a period of 6 weeks (June 2020 to July 2020). Throughout Africa, lockdown measures were generally applied during this period. The application was open only to Africans over the age of 18 years. As the movement restrictions and social distancing made it impossible to collect data during this period, an online approach was taken. Besides the large population of South Africa, five other countries in SSA were selected for this research, based on the lockdown situation (partial or total), as well as the availability of the African Nutrition Leadership Program (ANLP) alumni who volunteered to help with data collection. The selected countries were Cameroon in the Central part of Africa, Ethiopia and Kenya representing the Eastern region, Nigeria and Ghana to represent Western Africa.

#### Sampling procedure

An online snowball sampling method was used for this study. In fact, due to the limits in movement and social isolation, the questionnaire was distributed via an Internet survey that was provided to respondents electronically. A structured questionnaire was firstly designed. The prepared survey instrument was then converted into the online format using Google Forms (Alphabet, Inc., Mountain View, California, United States), and this was designed in a way that ensured respondents’ anonymity as no personal details or e-mail address were required. Additionally, for the French-speaking Cameroonians, the questionnaire was translated into a French version which was also circulated. The questionnaire was piloted on 25 people, and suggestions were considered. The final version (validated) was put online and delivered to people from various socio-economic classes via e-mail and social media platforms such as Facebook Messenger and WhatsApp. Individuals with Internet access comprised the study’s participants. All the participants in this research were required to be at least 18 years old (inclusion criteria) and also to understand either English or French (exclusion criteria) in order to be able to complete the form.

#### Questionnaire design and data collection

The survey instrument used in this study was created based on an assessment of existing literature on the issue. Specifically, it collected data on socio-economic factors, special food substances and drinks consumed to prevent COVID-19 during the lockdown and information sources on COVID-19 prevention. The online questionnaire was drafted and validated first before it was hosted online. Nutrition and health experts were asked to assess the instrument and give their view on the appropriateness of the survey questions. The questionnaire was pretested on 25 individuals in a pilot study, as previously mentioned, following which they were removed from the study. Expert advice was used to amend or improve the questionnaire into a simpler, more concise form that could be completed in about 7 min. Over a 6-week period, this study was conducted online. A target sample size of 800 was set and data collected online. This is a report of the collected 817 replies to the online survey on special diets consumed in SSA during the COVID-19 lockdown.

### Data analysis

For all statistical analyses in this study, the Statistical Package for Social Sciences (SPSS), version 25.0 (IBM Corporation, Armonk, New York, United States) was used. The proportions of responses were calculated using descriptive statistics (frequency charts, graphs). To analyse the association between sociodemographic characteristics of respondents and their dietary habits, the Pearson correlation test was used. Variables with *p* < 0.05 were considered significant at 95% confidence interval (CI).

### Informed consent and data privacy

Before completing the questionnaire, respondents were asked to carefully read and understand the summary of the research. The informed consent process assured survey participants that all information given was to be strictly used for research purposes. Participants’ responses were recorded anonymously and kept confidential according to Google’s privacy policy.^1^ Names and/or contact information were not required to participate in the study. Furthermore, they were allowed to stop participating in the study and leave the questionnaire page at any point before submission, and that way their responses would not be saved. The ‘submit’ button was only used for saving responses when tapped on. Participants voluntarily agreed to take part in this anonymous study by completing the survey.

### Ethical considerations

Ethical clearance to conduct this study was obtained from the School of Tourism and Hospitality Research Ethics Committee of the University of Johannesburg (ref. no. 20STH04).

## Results

### Sociodemographic characteristics of the sample population

Table 1 shows the sociodemographic characteristics of the surveyed population (817 people). In terms of country representation, Cameroon accounted for 30.1%, Ethiopia 4.3%, Ghana 7%, Kenya 10.6%, Nigeria 25.8% and South Africa 22.2%. Five races were represented in this population, with the majority being black people, followed by Indian-, white-, mixed race- and Asian people; 60.8% of them were women. A total of 47.9% of them were between 30 and 49 years of age, while 41.6% and 10.5% were older and younger, respectively. Across the board, most of the participants (93.5%) had a tertiary education, while few (6.4%) or less (0.1%) only had a secondary education. There were more single respondents (54%) than married respondents (47.8%). Twenty-one per cent of respondents lived in a household of 1–2 people, the rest in households of 3–5 people (56.7%) or more (22.3%). The majority worked in the public sector (32.6%), followed by private sector (40.4%), while a minority had informal jobs (8%) or were unemployed (27.1%). More than one-third (38%) of the study participants reported receiving between $100 and $300 as monthly income.

**TABLE 1:** Sociodemographic characteristics of study participants (*N* = 817).

Variable	Subvariable	Frequency (*N* = 817)	Percentage (100%)
Sex	Male	320	39.2
	Female	497	60.8
Age group (years)	18–29	340	41.6
	30–49	391	47.9
	50 and older	86	10.5
Country of residence	Cameroon	246	30.1
	Nigeria	211	25.8
	South Africa	181	22.2
	Ghana	57	7.0
	Kenya	87	10.6
	Ethiopia	35	4.3
Race	Black people	715	87.5
	Mixed race people	24	2.9
	Indian people	40	4.9
	White people	36	4.4
	Asian people	2	0.2
Marital status	Married	330	40.4
	Single	441	54
	Widow	15	1.8
	Concubine	8	1.0
	Divorced	23	2.8
Educational level	Primary school	1	0.1
	Secondary school	52	6.4
	University or tertiary institution	764	935
Employment sector	Informal	65	8.0
	Public (working for the government)	222	27.2
	Private (in a registered company or organisation)	309	37.8
	Unemployed	221	27.1
Economic status	Affluent (> $500)	134	16.4
	Above average ($301 – $500)	183	22.4
	Average ($100 – $300)	312	38.2
	Poor (< $100)	188	23.0
Household size	1–2 people	223	27.3
	3–5 people	386	47.2
	6 or more people	208	25.5

### Special substances consumed to prevent COVID-19 infection

Table 2 summarises the particular food substances that were consumed during the COVID-19 lockdown to prevent COVID-19 infection. Between 42.3% and 64.2% of respondents did not follow any specific diet in order to prevent COVID-19. The many types of unusual foods and drinks that were reported were grouped into six categories. ‘Infusions made of spices with or without honey’ was the most popular category, with garlic, lemons, limes, ginger and turmeric being the most commonly used ingredients. The highest and lowest consumers, respectively, were Cameroonians and South Africans. ‘Fruits and vegetables’ came in second, with lemons, limes, oranges, pineapples and apples being the most frequently mentioned. Kenyans and Ethiopians had the highest consumption rate (22.1%), considerably ahead of South Africans (11.6%), Nigerians and Ghanaians (11.4%) and Cameroonians (4.1%). ‘Bark infusions and decoctions’ of ‘ekuk’ (*Alstonia boonei*), ‘kinkeliba’ (*Combretum micranthum*) and ‘quinquina’ (*Cinchona officinalis*), as well as ‘maceration, decoction or infusion’ of neem leaves, artemisia, aloe vera or citronella, were two other groupings of medicinal plants. Cameroon was the only country where they were consumed (Central African region). ‘Local beverages’, such as hibiscus, beetroot or lemonade, made up the fifth category. About 4.1% were from Western Africa (Nigeria and Ghana), 0.8% were from Ethiopia and Kenya and the rest were from South Africa (0.6%). Those who used ‘conventional medicines or supplements’, particularly vitamin C and zinc, were placed in the last category. The majority of South African respondents (15.5%) reported this, followed by West African respondents (2.1%), and East African respondents (1.6%). Table 3 shows that gender, race, geographical region and monthly income were significant determinants of consumption of the special foods or drinks reported (*p* < 0.05). Women, respondents in South Africa, white people and the affluent used conventional medicines more often. The consumption of spice or plant infusions seemed more popular among Cameroonians and East Africans. In addition, fruit and vegetable consumption seemed to be higher in the East African countries (Ethiopia and Kenya).

**TABLE 2:** Special foods and drinks reported to be consumed by participants to prevent COVID-19.

Code	Category	Components	Percentage (%)			
			Cameroon (*N* = 246)	South Africa (*N* = 181)	Nigeria and Ghana (*N* = 268)	Ethiopia and Kenya (*N* = 122)
0	No preventive diets	-	42.3	51.4	64.2	43.4
1	Infusions made of spices, with or without honey	**Garlic, lemon, lime, ginger**, **turmeric**, hot pepper, cayenne pepper, black seeds; clove, celery sticks, curcuma	41.5	19.9	16.8	32.0
2	Plant macerations, infusions and decoctions	**Artemisia**, neem leaves, citronella, aloe vera	8.1	1.1	1.1	0.0
3	Herbal infusions (barks) and decoctions	**Kinkeliba (Combretum micranthum), quinquina (Cinchona officinalis)**, ekuk (Alstonia boonei), ebam (Picralima nitida)	3.3	0.0	0.0	0.0
4	Conventional medicines	**Cal-C-Vita, vitamin C**, vitamin B, **Efferflu C, zinc**, vitamin D	0.0	15.5	2.6	1.6
5	Beverages or drinks	**Bissap (hibiscus) drink,** beetroot juice, Phyllanthus drink, lemonade	0.0	0.6	4.1	0.8
6	Fruits and vegetables	**Lemon, orange**, apple, **pineapple**, pear, mango, pawpaw, berries	4.9	11.6	11.2	22.1

Note: Bold text indicates most used or consumed.

**TABLE 3:** Association between special diets or drinks consumed and respondents’ sociodemographic characteristics.

Variable	Special foods or drinks category code (%)						Total (%)	Pearson chi-square		
	0	1	2+3	4	5	6		Value	*df*	Asymptotic significance
**Country of residence**								197 937†	18	0
Cameroon (Central)	42.30	41.50	11.40	0.00	0.00	4.90	100.00	-	-	-
Nigeria and Ghana(West)	64.20	16.80	1.10	2.60	4.10	11.20	100.00	-	-	-
South Africa (South)	51.40	19.90	1.10	15.50	0.60	11.60	100.00	-	-	-
Ethiopia and Kenya(East)	43.40	32.00	0.00	1.60	0.80	22.10	100.00	-	-	-
**Gender**								19 760†	6	0.003
Male	56.60	24.40	6.30	1.90	1.30	9.70	100.00	-	-	-
Female	48.50	29.00	2.60	6.20	1.80	11.90	100.00	-	-	-
**Race**								39 605†	24	0.024
Black people	50.90	29.00	4.50	3.20	1.70	10.80	100.00	-	-	-
Mixed race people	45.80	20.80	4.20	12.50	0.00	16.70	100.00	-	-	-
Indian people	57.50	17.50	0.00	12.50	0.00	12.50	100.00	-	-	-
White people	61.10	8.30	0.00	16.70	2.80	11.10	100.00	-	-	-
Asian people	100.00	0.00	0.00	0.00	0.00	0.00	100.00	-	-	-
**Age group**								11 809†	12	0.461
18–29	52.10	27.90	5.00	3.80	1.20	10.00	100.00	-	-	-
30–49	50.40	27.90	4.10	4.30	1.50	11.80	100.00	-	-	-
50 and older	55.80	20.90	0.00	8.10	3.50	11.60	100.00	-	-	-
**Educational level**								4 828†	12	0.963
Primary school	100.00	0.00	0.00	0.00	0.00	0.00	100.00	-	-	-
Secondary school	46.20	28.80	3.80	1.90	1.90	17.30	100.00	-	-	-
University or tertiary	52.00	27.10	4.00	4.70	1.60	10.60	100.00	-	-	-
**Marital status**								30 582†	24	0.166
Concubine	62.50	37.50	0.00	0.00	0.00	0.00	100.00	-	-	-
Divorced	52.20	8.70	0.00	21.70	0.00	17.40	100.00	-	-	-
Married	51.80	28.20	3.30	3.60	2.10	10.90	100.00	-	-	-
Single	51.20	26.50	5.00	4.50	1.40	11.30	100.00	-	-	-
Widow	53.30	46.70	0.00	0.00	0.00	0.00	100.00	-	-	-
**Household size**								19 805†	12	0.071
1–2 people	52.90	25.60	5.80	4.00	1.30	10.30	100.00	-	-	-
3–5 people	52.30	24.40	3.30	6.00	1.30	12.70	100.00	-	-	-
6 or more	49.00	34.10	3.30	2.40	2.40	8.70	100.00	-	-	-
**Employment status**								26 361†	18	0.092
Informal	67.70	18.50	6.20	0.00	0.00	7.70	100.00	-	-	-
Private (for a registered company or organisations)	54.00	26.20	3.20	4.90	1.60	10.00	100.00	-	-	-
Public (working for the government)	47.70	26.10	3.70	5.00	2.70	14.90	100.00	-	-	-
Unemployed	47.50	32.10	5.00	5.00	0.90	9.50	100.00	-	-	-
**Monthly income**								39 647†	18	0.002
Affluent (> $500)	55.20	24.60	0.70	8.20	0.70	10.40	100.00	-	-	-
Above average ($301 – $500)	49.20	25.70	2.10	5.50	3.30	14.20	100.00	-	-	-
Average ($100 – $300)	50.60	26.60	4.80	3.20	1.60	13.10	100.00	-	-	-
Poor (< $100)	53.20	31.40	6.90	3.20	0.50	4.80	100.00	-	-	-

Note: Significance accepted at *p* < 0.05.

0, none; 1, infusion of spices or herbs; 2+3, plant decoctions; 4, conventional medicines; 5, local beverages; 6, fruits and vegetables.

### Reported sources of information and reasons for consumption of COVID-19 preventives

Table 4 shows the respondents’ reported sources of information on COVID-19 preventive diets, as well as their perceived motivations for consuming them. Social media (external information on social media uploaded by nonrelatives – 45%), friends or relatives (42%), radio or TV (28%) and culture or tradition (21%) were the most generally reported sources of knowledge. The least reported source of information on COVID-19 preventive diets was lectures (12%). According to the results presented, many respondents (70%) consumed these preventive foods or drinks in order to boost their immunity; another reason given was to protect the body (28%). In addition, 22% of people reported that these special diets had the capability of detoxifying the body, ejecting harmful substances out of the body.

**TABLE 4:** Reported sources of information on COVID-19 prevention.

Query	Responses	%
Reported sources for information on COVID-19 preventive diets	Social media (e.g. Facebook)	45.15
	Friends or relatives	42.18
	Radio or television	28.25
	Culture or tradition	21.00
	Lectures	12.00
Reasons given for consumption of COVID-19 preventives	Boosts immunity	70.25
	Protects the body	28.05
	Detoxifies the body	22.30
	Kills the virus	12.00
	Prevents the virus from entering the body	11.90
	Do not know	12.30

## Discussion

The results of this study are in accordance with the report by Panyod, Ho and Sheen (2020), which:

[*S*]upported the consumption of foods and herbs as dietary or complementary therapy to prevent infection because existing evidence suggests that certain foods and herbs may be antiviral against SARS-Cov-2 and can also prevent COVID-19. (p. 420)

With regard to the reported special diets, fruits, vegetables, herbs and spices are natural sources of vitamins and minerals, and it is commonly advised that people consume them regularly to stay healthy. The consumption of these plant source preventive substances has been promoted globally because they are natural sources of vitamin C, vitamin D, zinc and selenium, which have been of interest in the fight against the COVID-19 virus. Researchers report that nutrients, zinc and vitamin D prevent the spread of SARS-CoV-2 viruses; vitamin C boosts the immune system, and selenium boosts a person’s antioxidant defences in the body (Carr & Maggini 2017; Kumar et al. 2020; Martineau et al. 2019). Hibiscus is high in vitamin C and iron, helps to enhance the body’s defence system and has a variety of other nutritional benefits (Riaz & Chopra 2018). In the fight against viral infections that cause cough or cold, infusions made of ginger, garlic, or lime with honey have been reported to be helpful (Raal et al. 2013). Plants such as kinkeliba and quinquina, on the other hand, are useful and natural sources of chloroquine (Ngene et al. 2015), an active ingredient which is used to treat COVID-19 in numerous countries (Lawal 2021). All of the unique foods or drinks consumed in reaction to the COVID-19 pandemic may have been effective to a certain level (Rahman, Mosaddik & Alam 2021) and may have contributed to the relatively lower COVID-19 morbidity or mortality rates in SSA (Lawal 2021; Tcheutchoua et al. 2020). Carrying out a systematic review of their properties could help to prioritise these preventive food substances. Furthermore, the high percentage of people who do not consume the reported special foods and drinks (in some countries like SA) suggests that dietary practices and nutrition-based interventions could play a key role in COVID-19 prevention and severity reduction (Onyenweaku, Kesa & Akah 2022). In addition, good health care facilities and adherence to the COVID-19 regulations such as physical distancing, handwashing and wearing of face masks can also go a long way in reducing the incidence rate of COVID-19.

Almost all educational institutions were closed during the 2020 COVID-19 lockdown; therefore, the handful who chose the ‘lectures’ option as a source of COVID-19 information may be referring to courses or programmes hosted online, which became very popular and were also effective during and after the lockdown and social-distancing periods. Recently, there has been an increase in social media platforms such as Facebook, Twitter, WhatsApp and Instagram, courtesy of technological innovations. These platforms have been instrumental in disseminating information and increasing awareness of current issues. Many useful data have been generated and information shared via these platforms, especially about how to stay safe and strong during the COVID-19 pandemic. In turn, this has significantly contributed towards decreasing the spread of the virus and has also discouraged the public from accepting various misconceptions about the pandemic.

In addition to these results and new advancements, some dietary advice for managing the pandemic has been provided based on scientific study. Seventy per cent of the publications collated in a review by De Faria Coelho-Ravagnani et al. (2021) promoted the consumption of fruits, vegetables and whole grain diets.

Two nutrition societies, from Italy and Spain (Academia Española de Nutrición y Dietética (la Academia) y el Consejo General de Colegios Oficiales de Dietistas-Nutricionistas (2020) is the source and is in the reference list, as well as Società Italiana Di Nutrizione Umana (2020), have suggested a minimum of 5 servings of fruits and vegetables each day.

Fruits and vegetables are high in vitamins and minerals, such as vitamins A, C, D, E and B complex, as well as zinc and selenium, which are significant immune system modulators (Maggini, Pierre & Calder 2018). Micronutrients play a role in both innate and adaptive immunological responses, contributing to immune function through a variety of mechanisms. Vitamins A, C, D, E, B_6_ and B_12_, as well as zinc and selenium, aid the adaptive immune response by influencing T- and B-cell development, proliferation and normal function; they also regulate antibody synthesis and function (Gombart, Pierre & Maggini 2020), enhance cell-mediated immunity and assist in the recognition and destruction of pathogens. Furthermore, they have antimicrobial properties and modulate in inflammation (Gombart et al. 2020).

In like manner, many nutrition bodies recommend limiting salt, fat and sugar consumption and meat portions, as well as other animal products, to reduce saturated fat consumption (Academia Española de Nutrición y Dietética [la Academia] y el Consejo General de Colegios Oficiales de Dietistas-Nutricionistas 2020; De Faria Coelho-Ravagnani et al. 2021). De Faria Coelho-Ravagnani et al. (2021) recommend incorporating low-fat dairy products and healthy fats, such as olive oil and fish oil, into the diet. Moreover, they recommend sauces, spices and herbs as salt alternatives (Dietitians Australia Website 2022). Low-grade inflammation is favoured by high saturated fat intake and should be avoided (Ruiz-Nuñez, Dijck-Brouwer & Muskiet 2016). On the other hand, mono and poly unsaturated fatty acids are reported to possess favourable immune-modulatory action (Hunsche, Hernandez & Gheorghe 2018). According to De Faria Coelho-Ravagnani et al. (2020), drinking water or maintaining adequate hydration is also recommended, but there is no guidance on quantity or volume. Water is required for many *in vivo* functions, namely body temperature control, headache prevention, cellular homeostasis, cognitive function, mood regulation, gastrointestinal, kidney and heart function (El-Sharkawy, Sahota & Lobo 2015).

Studies of probiotics (of the Lactobacillus and Bifidobacterium genera) have shown promising outcomes in the aspect of increased immunity; hence, the use of probiotics has also been recommended (Azad, Sarker & Li 2018). Similarly, the Food and Agriculture Organization of the United Nations (FAO 2020) recommends that alcohol intake be limited, but no specific amounts are stipulated. Elevated levels of alcohol intake increase the risk of tuberculosis and bacterial and viral pneumonia in humans and animals, and they also decrease the host’s immunity to viral infections (Szabo & Mandrekar 2009).

Research also suggests the possibility of using supplements to meet dietary recommendations (Associação Brasileira de Nutrologia 2020; European Food Information Council Website 2020). It was reported by the Brazilian Association of Clinical Nutrition (Associação Brasileira de Nutrologia 2020) that supplementation of ascorbic acid may be beneficial for individuals prone to viral respiratory infections. A well-known antioxidant, vitamin C can promote chemotaxis, phagocytosis, reactive oxygen species production and, finally, microbial elimination (Carr & Maggini 2017). Likewise, selenium and zinc are antioxidant micronutrients frequently recommended for supplementation because they both inhibit oxidative stress (Associação Brasileira de Nutrologia 2020).

In summary, foods and drinks are often a preferred source of antioxidants instead of dietary supplements. However, supplements should be considered mainly by individuals who struggle to meet their daily dietary requirements, and dietary supplements are not to be solely relied on for COVID-19 prevention. Further studies on the effects of micronutrient supplementation in COVID-19 related outcomes (such as disease severity, recovery rate, death, etc.) will be useful.

## Conclusion

The results of the online cross-sectional survey revealed a diversity of special food and drinks consumed by SSAs to prevent COVID-19, with some of them having been scientifically confirmed as boosting the body’s immune system. Neem leaves, artemisia, vitamin C and zinc supplements and infusions made with lemon, ginger and garlic were some of the most commonly consumed preventive foods and drinks in the study participants. However, it was observed that those in South Africa (likewise Nigeria and Ghana) did not consume as many of these useful foods and drinks with prophylactic properties when compared to the other countries, which consumed a variety of these plant substances. There is an important need to promote the consumption of these preventive foods and drinks which are natural and healthy; their toxicity levels should also be established in order to eliminate risks or adverse side effects that could be caused by excessive intake.

### Limitations of the study

The COVID-19 lockdowns limited this research to an online survey, as the researchers were unable to travel to certain rural or semi-urban settings to include individuals from those areas. As a result, this research is limited to a specific socio-economic group of people who are well-educated and have access to Android phones and the Internet. The validity of responses is a common issue with online surveys, and it can be difficult to determine, especially because responses are dependent on participants’ impressions.

## Footnotes

1 For more information, see https://policies.google.com/privacy?hl=en
